# The Global Case-Fatality Rate of COVID-19 Has Been Declining Since May 2020

**DOI:** 10.4269/ajtmh.20-1496

**Published:** 2021-04-21

**Authors:** Mohammad Nayeem Hasan, Najmul Haider, Florian L. Stigler, Rumi Ahmed Khan, David McCoy, Alimuddin Zumla, Richard A. Kock, Md. Jamal Uddin

**Affiliations:** 1 Department of Statistics, Shahjalal University of Science and Technology, Sylhet 3114, Bangladesh;; 2 The Royal Veterinary College, University of London, Hawkshead Lane, North Mymms, Hatfield, Hertfordshire, United Kingdom;; 3 Austrian Sickness Fund, Vienna, Austria;; 4 Department of Critical Care Medicine, Orlando Regional Medical Centre, Orlando, Florida;; 5 Institute of Population Health Sciences, Barts and London Medical and Dental School, Queen Mary University of London, London, United Kingdom;; 6 Department of Infection, Division of Infection and Immunity, Centre for Clinical Microbiology, Royal Free Campus, University College London, London, United Kingdom;; 7 National Institute for Health Research Biomedical Research Centre, University College London Hospitals, London, United Kingdom

## Abstract

The objective of this study was to evaluate the trend of reported case fatality rate (rCFR) of COVID-19 over time, using globally reported COVID-19 cases and mortality data. We collected daily COVID-19 diagnoses and mortality data from the WHO’s daily situation reports dated January 1 to December 31, 2020. We performed three time-series models [simple exponential smoothing, auto-regressive integrated moving average, and automatic forecasting time-series (Prophet)] to identify the global trend of rCFR for COVID-19. We used beta regression models to investigate the association between the rCFR and potential predictors of each country and reported incidence rate ratios (IRRs) of each variable. The weekly global cumulative COVID-19 rCFR reached a peak at 7.23% during the 17th week (April 22–28, 2020). We found a positive and increasing trend for global daily rCFR values of COVID-19 until the 17th week (pre-peak period) and then a strong declining trend up until the 53rd week (post-peak period) toward 2.2% (December 29–31, 2020). In pre-peak of rCFR, the percentage of people aged 65 and above and the prevalence of obesity were significantly associated with the COVID-19 rCFR. The declining trend of global COVID-19 rCFR was not merely because of increased COVID-19 testing, because COVID-19 tests per 1,000 population had poor predictive value. Decreasing rCFR could be explained by an increased rate of infection in younger people or by the improvement of health care management, shielding from infection, and/or repurposing of several drugs that had shown a beneficial effect on reducing fatality because of COVID-19.

## INTRODUCTION

On March 11, 2020, the World Health Organization (WHO) declared the coronavirus disease 2019 (COVID-19) outbreak as a global pandemic.^1^ As of January 31, 2020, there are over 100 million identified cases and 2.1 million deaths of COVID-19 reported worldwide in 213 countries and territories.^2^ The case fatality rate (CFR) of COVID-19, which is defined as the proportion of death because of a specific disease among those diagnosed with it, varies greatly in different countries. For example, the CFR of COVID-19 varies from 28.9% in Yemen to 1% in Singapore and Qatar^3,4^ as of December 31, 2020. Several studies described possible drivers behind such national-level variation.^3,5,6^ According to the study by Liang et al. (2020), the mortality rate of COVID-19 is negatively associated with COVID-19 test number per 100 people, government effectiveness score, and the number of hospital beds.^3^ The study further showed a positive correlation between the proportion of the population aged 65 years and above among those being infected and the transport infrastructure quality score.^3^ Individual patient-level data showed that CFR can be strongly explained by age, but also by obesity and underlying diseases, for example, coronary heart disease, diabetes, and hypertension.^7,8^ However, little is reported about how the CFR has changed globally over time.

As the pandemic is progressing, the countries are gaining experience and building capacity to manage the severity of COVID-19. A few drugs (e.g., dexamethasone, tocilizumab, and sarilumab^9,10^) had shown some degree of effectiveness in reducing deaths or hospital stays of COVID-19 patients. There are some recent data suggesting that aggressive thromboprophylaxis or even empiric use of full anticoagulation in mechanically ventilated COVID-19 patients contributes to recovery in some cases.^11^ Testing capacity has increased in most countries of the world over time, and that is being useful in the detection of asymptomatic and mild cases. Thus, this is important to quantify whether the CFR of COVID-19 has changed over time. The objective of this study was to examine the variation of reported CFR of COVID-19 based on reported COVID-19 cases and mortality data globally over time and to identify variables that could potentially explain these differences in the CFR of the COVID-19 pandemic.

## METHODS

We used three forecasting models [i.e., simple exponential smoothing (SES), auto-regressive integrated moving average (ARIMA), and automatic time-series forecasting models] to identify the global trend of rCFR for COVID-19. Second, we used the Mann–Kendall (M–K) trend analysis to identify existence of any trend and the direction of the trend (increasing or decreasing). Finally, we developed a beta-regression model of explanatory variables to identify whether the variables have any relationship between the country’s rCFR of COVID-19. All these three different approaches helped us to make a plausible conclusion on the global trend of COVID-19 CFR and factors affecting the CFR of COVID-19 in different phases of the pandemic. All analyses were carried out using the statistical software R, version 3.5.2.2.

### COVID-19 data.

The necessary COVID-19 related data, including daily new cases, daily new deaths, total deaths, total deaths per million, and total cases from the WHO daily COVID-19 situation reports of 210 countries were collected from January 1 to December 31, 2020. The ARIMA, SES, and Prophet models were fitted for the full dataset.^12^

### Reported case-fatality rate (rCFR).

We estimated cumulative rCFR COVID-19 as the number of deaths per 100 COVID-19 confirmed cases. Because the number of cases and deaths both are a fraction of total cases or deaths, we considered the term as reported CFR or simply as rCFR.^13^

### Time series model to predict the trend.

We performed three time-series models, including SES, ARIMA, and Prophet, to identify the global trend of rCFR for COVID-19. We selected all these time series models because the outcome variable (cumulative rCFR) is dependent on the previous records and all these three models can take this into account. Using the time series models with the reported COVID-19 data, we forecasted trends for the prospective 10 days and visualizing in the figure. SES was used as a benchmark to compare the performance of the ARIMA and Prophet models. We also used M–K trend analysis to identify the daily or weekly cumulative trend (increasing or decreasing) of COVID-19 rCFR.

### Simple exponential smoothing.

Simple exponential smoothing is one of the familiar methods for forecasting procedures.^14^ The SES is a short-term forecasting model that assumes data fluctuates around a relatively stable mean.^15^ For infectious diseases in general, this method has been shown to be reasonably accurate and reliable.^16–18^ It takes into account the more recent observations and exponentially reduces the weights of older observations.^19^ The SES model for this study had been carried out using R package *fpp2*.^20^

### Auto-regressive integrated moving average (ARIMA).

We performed an ARIMA model to forecast the trend of global weekly cumulative rCFR. The ARIMA model is an exploratory, data-oriented method that allows the user to fit an appropriate model adapted from the structure of the data itself.^21^ This model assumes that the time series values are linearly related and intends to extract local patterns by eliminating high-frequency noise from the data.^22^

The benefit of ARIMA models is the ability to adjust to dynamically oriented systems that evolve over time by updating the model to forecast the system’s future state based on recent events.^23^ The ARIMA model for this study had been carried out using R package *forecast*.^24^

### Automatic forecasting time-series model (Prophet).

We also performed a decomposable automatic forecasting time-series model called Prophet using R package *prophet* to predict the 10-day fatality rate and to compare it with rCFR.^25^ The Prophet model ignores the temporal dependence of the data. Moreover, the irregular observations are allowed in the data set, and the model fits very quickly.^26^ It is also robust for missing data and generally manages outliers well.^27^ There are three main features of the model, i.e., trend, seasonality, and holidays. It can be represented as

$$\mathrm{Y (t) =}\text{g}\mathrm{(t) + s(t) + h(t) +}\in_{t}$$

where the model parameters *g(t), s(t), h(t), ∈*_*t*_ are a piecewise linear curve for modeling nonperiodic changes in time series, periodic changes, and the effects of holidays with irregular schedules considered in the model by some parameters, respectively. The error term accounts for any unexpected changes for which the model does not account.^27^

### Mann–Kendall (M–K) trend.

We used weekly cumulative rCFR data and performed the M–K trend test to identify the trend of COVID-19 rCFR for both the pre-peak and post-peak period.^28^

The M–K method is a nonparametric test that provides an indicator of whether there is a monotonous trend and whether there is a positive or negative trend.^28^ The M–K test statistic is robust when dealing with non-normally distributed data, censored data, and time series with missing values because it is calculated by ranks and sequences of time series rather than the original values.^29^

In addition, the Sen’s slope test was applied to determine the changes in COVID-19 rCFR in both periods.^30^ M–K and Sen’s slope trend analysis had been carried out using R package *trend*.^31^

### Empirical evaluation.

The ARIMA and Prophet models are empirically assessed by comparing their results to benchmarks in predicting the rCFR. This benchmark permitted us to assess the performance gains made by their counterparts.^32^ The SES also allows the most appropriate nonseasonal model for each series, allowing for any kind of error or trend component. Then, we analyzed and compared the performance of the studied time series models with some of the commonly used measures to evaluate the prediction significance, including coefficient of determination (*R*^2^), root mean square error (RMSE), and mean absolute error (MAE).

### Outcome and predictor variables.

We used rCFR as the outcome variable; we also collected and used several predictors data from the World Bank and other UN sources, such as population density,^33^ percentage of people above 65 years of age,^34^ Gross Domestic Product (GDP),^35^ worldwide governance indicators (WGI),^36^ and Global Health Security Index (GHSI),^37^ the prevalence of obesity^38^ in our analyses. We also included country-specific prevalence of diabetes and cardiovascular disease to explain the variation of COVID-19 rCFR. The GHSI index scored between 0 and 100 to indicate the country’s capacity for early detection and reporting for epidemics.^37^ The WGI scored between −2.5 and 2.5, where −2.5 indicates the weakest and 2.5 indicates the strongest governance performance.^36^ The median age of the diagnosed people (daily) is an important variable that we could not include in the model because these data are not publicly available for most countries of the world.

### Statistical analysis.

**Figure 1.**
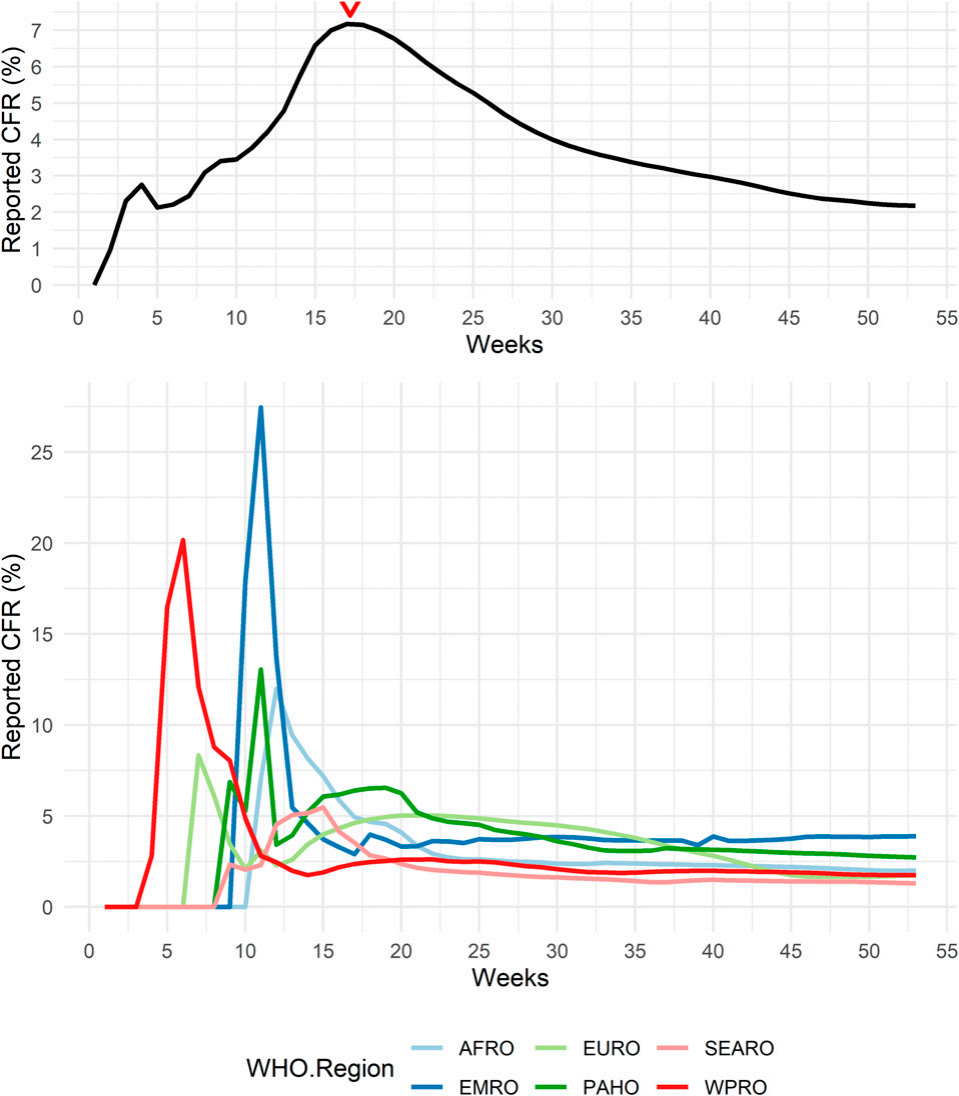
The changes of global weekly cumulative reported case-fatality rate (rCFR) of COVID-19 (top) with the weekly cumulative rCFR in different WHO regions. The peak is observed in the 17th epidemiological week (April 22–28), which is also dominated by WHO regions PAHO, EMRO, and EURO). AFRO = African Region; PAHO = Region of the Americas; SEARO = South-East Asia Region; EURO = European Region; EMRO = Eastern Mediterranean Region; WPRO = Western Pacific Region. This figure appears in color at www.ajtmh.org.

We observed that the rCFR of COVID-19 has changed over time (Figure 1). We also observed the rCFR reached a peak at the 17th epidemiological week (April 22–28, 2020, considering January 1, 2020 as the start of epidemiological week) and then the trend started to decline. Using a time-series model alone would not allow us to identify the reason behind the increasing and decreasing trend of COVID-19 rCFR. We explored whether the relationship between the rCFR of COVID-19 and country-level explanatory variables vary over time or if they remain the same in two periods through a regression model. We divided the dataset into two halves: one until it reaches a peak (the first to 17th weeks), called “before peak rCFR” or simply “pre-peak period” and another with the 18th to 53rd weeks (December 29–31, 2020), called “after peak rCFR period” or simply as “post-peak period.” Because the trend of rCFR in both periods is different, we ran a beta regression model separately for each dataset to investigate the association between possible explanatory variables, and we explored which variables affected the most in both periods separately.

### Beta regression models.

As the outcome variable (rCFR) varies in an interval of 0 or 1, we used beta regression models to look at the association between possible explanatory variables and the rCFR.^39,40^ We applied beta-regression model^41^ of explanatory variables of two different periods (pre and post peak). We reported incidence rate ratios (IRRs) after adjusting them for population density (per square kilometer), the percentage of people above 65 years of age of the total population, the prevalence of obesity in the country, total test per thousand, GHSI, GDP (per million), and WGI, with 95% confidence intervals (CIs). We also adjusted for the stage of the epidemic in each country by including a variable of interval (in days) between detection of the first COVID-19 case in the country and the last date of data collection (April 28 for the pre-peak period and December 31 for the post-peak period). We used the variance inflation factor (VIF) value to examine multicollinearity in the dataset with a cut-off value of 5,^42^ and thus we discarded variables from our model those that showed multicollinearity (prevalence of diabetes and cardiovascular disease in the country). The beta regression models for this study had been carried out using R package *betareg*.^31^

**Figure 2.**
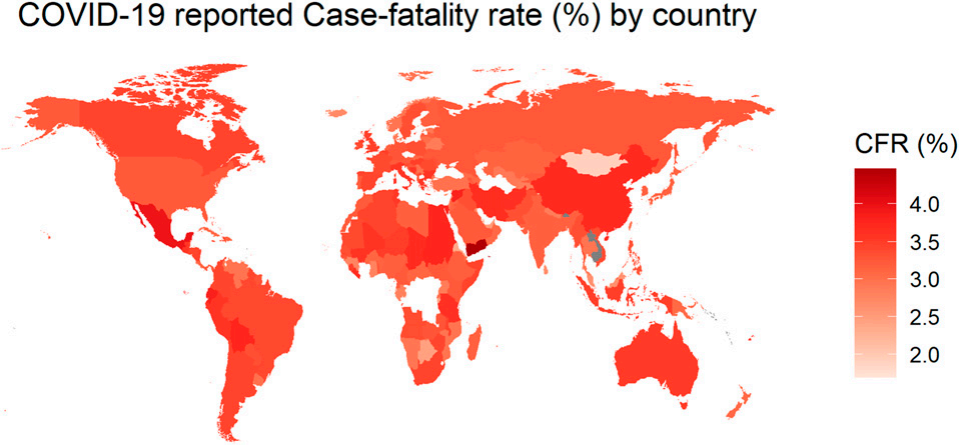
The reported case-fatality rate (rCFR) of COVID-19 in different countries or territories of the world, January 1, 2020 to December 31, 2020 (data in log scale). This figure appears in color at www.ajtmh.org.

We also plotted the estimated weekly cumulative rCFR of COVID-19 globally and for different WHO regions (Figure 1). WHO member states are grouped into six WHO regions: African Region (AFRO), Region of the Americas (PAHO), South-East Asia Region (SEARO), European Region (EURO), Eastern Mediterranean Region (EMRO), and Western Pacific Region (WPRO), indicating the early spread of the virus in WPRO before the others.^43^ The EU countries consist of Austria, Belgium, Bulgaria, Croatia, Republic of Cyprus, Czech Republic, Denmark, Estonia, Finland, France, Germany, Greece, Hungary, Ireland, Italy, Latvia, Lithuania, Luxembourg, Malta, Netherlands, Poland, Portugal, Romania, Slovakia, Slovenia, Spain, and Sweden. We mapped the global cumulative rCFR of COVID-19 (Figure 2).

Finally, we aimed to plot the rate of COVID-19 infection in the different age groups over time. However, age-specific infection data from most countries of the world are not publicly available. We could collect such data in detail from Germany and thus presented the changes of infection rate in different age group in Germany as an example^44^ (Supplemental Figure 2). Furthermore, we plotted the monthly global number of reported COVID-19 cases and deaths (Supplemental Figure 3).

## RESULTS

More than 96.9 million cumulative confirmed cases and 2.08 million deaths had been documented globally, and the global rCFR of COVID-19 was reported as 2.2% as of December 31, 2020. The weekly global cumulative rCFR of COVID-19 reached a peak at 7.23% during the 17th epidemiological week (April 22–28, 2020). The top five countries with COVID-19 rCFR are Yemen (28.9%), Italy (13.2%), United Kingdom (12.4%), Belgium (11.6%), and France (11.0%) (Figure 2 and Supplemental Figure 1). The weekly mean cumulative rCFR was 3.6% (95% CI: 2.5–4.6) for the pre-peak period and 3.8% (95% CI: 3.3–4.3) for the post-peak period. The peak of the global COVID-19 rCFR was dominated by different WHO regions, particularly at PAHO (especially the USA), EURO (especially the UK), and EMRO (especially Iran) (Figure 1). After the 17th week, the weekly cumulative rCFR declines gradually in most WHO regions.

**Figure 3.**
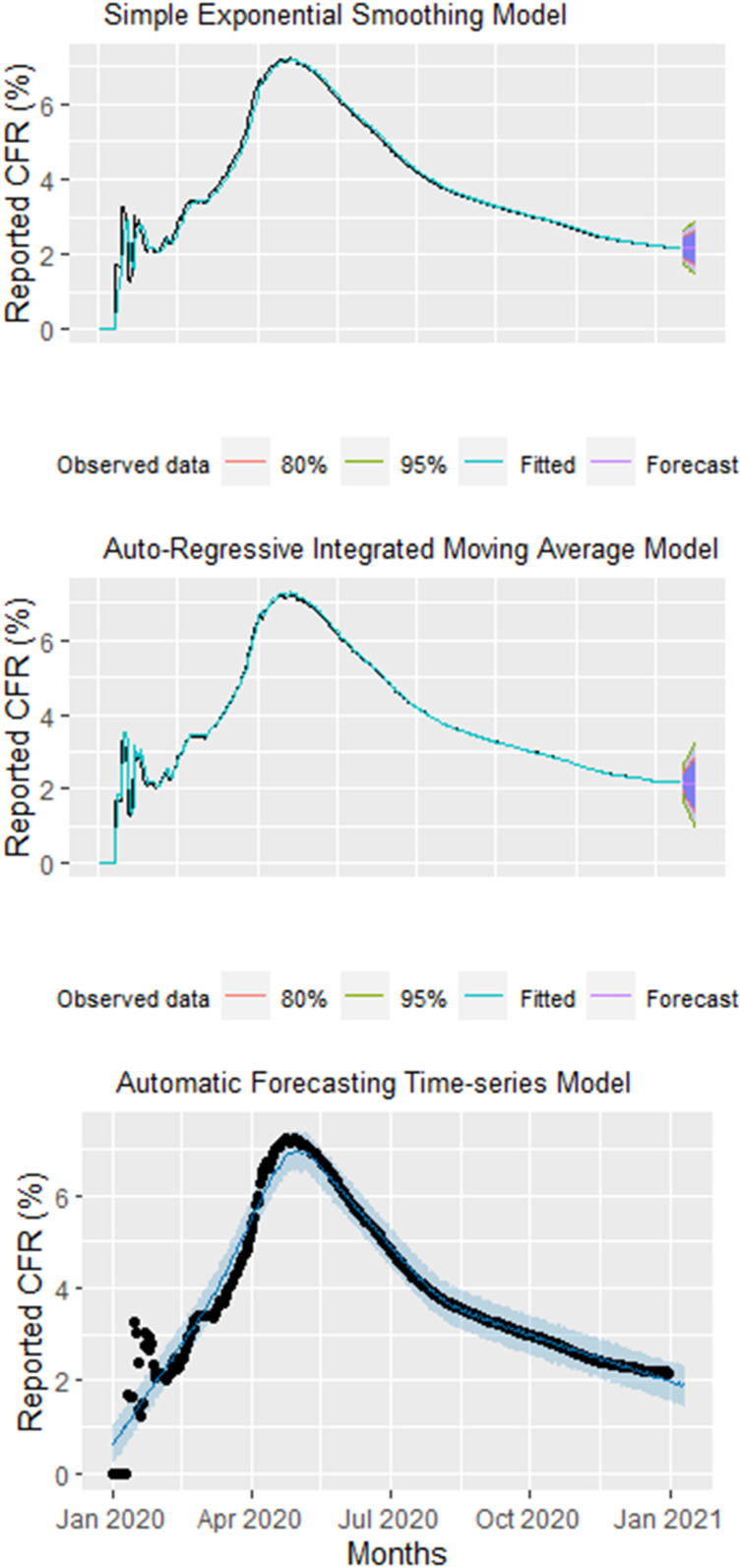
Top: Observed and predicted daily worldwide daily reported case-fatality rate (rCFR) using a simple exponential smoothing (SES) model. Middle: Observed and predicted daily worldwide daily cumulative rCFR using an auto-regressive integrated moving average (ARIMA) model. Bottom: Observed and predicted daily worldwide daily cumulative rCFR using an automatic forecasting time-series model (Prophet). Black dots = observed data; the blue line = predictive CFR; the shaded area = 95% confidence interval of predicted CFR. This figure appears in color at www.ajtmh.org.

In the SES model, we found a constant trend between observed and predictive global rCFR of COVID-19 with the *R*^2^, RMSE, and MAE being 98.17%, 0.23, and 0.11, respectively (Table 1 and Figure 3). In the ARIMA and Prophet models, we found a strong declining trend between observed and predictive global rCFR of COVID-19 with a *R*^2^, RMSE, and MAE value of 98.98% and 96.26%, 0.17 and 0.33, and 0.05 and 0.18, respectively (Table 1). In terms of accuracy, the ARIMA model performed better over the Prophet and SES models (with better *R*^2^, RMSE, and MAE values). The coefficient of determination of the ARIMA model was the larger, and errors are lower than the Prophet and benchmark SES models. According to the forecast in both models, the ratio of COVID-19 rCFR is expected to decrease considerably in the coming 10 days. The forecasting of global cumulative rCFR of COVID-19 for each model are shown in Figure 3.

**Table 1:** The summary of SES, ARIMA, automatic forecasting time-series model (Prophet), M–K trend, and Sen’s slope analysis

Method & Period	R^2^	RMSE	MAE
Simple exponential smoothing			
Overall	98.17%	0.23	0.11
			
Auto-regressive integrated moving average			
Overall ARIMA (0,2,1)	98.98%	0.17	0.05
			
Automatic forecasting time-series model			
Overall	96.26%	0.33	0.18

Mann–Kendall trend analysis	tau	*P*	
Before peak*	0.93	< 0.001	
After peak†	−1.0	< 0.001	

Sen’s slop test	Sen’s Slope	95% CI	
Before peak*	0.39	0.32 to 0.45	
After peak†	−0.12	−0.15 to −0.10	

ARIMA = auto-regressive integrated moving average; CFR = case fatality rate; M–K = Mann–Kendall; rCFR = reported case-fatality rate; MAE = mean absolute error; RMSE = root mean square error; SES = simple exponential smoothing. Prophet is the automatic forecasting time-series model. The SES, ARIMA, and Prophet models used daily cumulative CFR data whereas the M–K trend analysis and Sen’s slop used weekly cumulative CFR data. The Kendall’s Tau value permits a comparison of the strength of correlation between two data series (here, week of the year 2020 and rCFR).^28^

* Before peak = COVID-19 data from first week to 17th week (April 22–28, 2020).

† After peak = COVID-19 data from 18th week (after peak week) to 53rd week (December 29–31, 2020).

In an M–K trend analysis, we identified an increasing trend of cumulative rCFR for the week first to 17th (*P* < 0.001 and tau = 0.93). Using Sen’s slope test, we found that over the 17 weeks, the slope was 0.39 (95% CI: 0.32 to 0.45). We found a negative trend of cumulative rCFR for the period of weeks 18 to 53 (*P* < 0.001 and tau = −1.00). In Sen’s slop test, over the 36 weeks, the slope was –0.12 (95% CI: −0.15 to −0.10) (Table 1).

In the beta regression model for both epidemiological periods (pre- and post-peak period), the percentage of people aged 65 years or above the age of the population of the country (IRR: 1.05, 95% CI: 1.02–1.08 and 1.01 [0.98–1.03], respectively) and population density (IRR: 1.01 [1.01–1.02] and 1.01 [1.01–1.02], respectively) were significantly positively associated with COVID-19 rCFR (Table 2). The COVID-19 total tests per 1,000 was slightly negatively associated with the COVID-19 rCFR in both pre- and post-peak periods (0.98 [0.98–0.99] and 0.99 [0.98–1.01], respectively) (Table 2). Other variables significant in the pre-peak period were the prevalence of obesity, GDP, and WGI; and at the post-peak period were WGI, GDP, and GHSI (Table 2).

**Table 2:** Factors associated with rCFR of COVID-19 using beta regression model

Variables	Before peak*			After peak†		
	IRR	95% CI	*P* value	IRR	95% CI	*P* value
The percentage of people aged 65 and above	**1.05**	**1.02–1.08**	**< 0.001**	1.01	0.98–1.03	0.749
Population density	**1.01**	**1.01**–**1.02**	**0.006**	**1.01**	**1.01**–**1.02**	**0.028**
COVID-19 total tests (/1000)	**0.98**	**0.98**–**0.99**	**0.002**	0.99	0.98–1.01	0.144
GHSI	1.01	0.98–1.02	0.778	**1.03**	**1.01**–**1.05**	**0.002**
GDP	**1.01**	**1.01**–**1.02**	**< 0.001**	**1.01**	**1.01**–**1.02**	**< 0.001**
WGI	**0.54**	**0.45**–**0.65**	**< 0.001**	**0.63**	**0.49**–**0.82**	**< 0.001**
Obesity (%)	**1.01**	**1.01**–**1.03**	**0.031**	1.01	1.00–1.03	0.104
Adjusted pseudo-*R*^2^	0.54			0.37		

CI = confidence interval; GDP = Gross Domestic Product; GHSI = Global Health Security Index; IRR = incidence rate ratio; rCFR = reported case fatality rate; WGI = Worldwide Governance Indicators. The IRR of 1.05 for the “percentage of people aged 65 and above” indicates that countries with 1% additional people ≥ 65 years old have an increased risk of rCFR by 5%. The data were collected for the dates of April 26, 2020 for the pre-peak period and December 31, 2020 for post-peak period. The values in bold letter indicate significant at 5% level.

* Before peak = COVID-19 data from 1st week to 17th week (April 22–28, 2020).

† After peak = COVID-19 data from 18th week (after peak week) to 53rd week (December 29–31, 2020).

Finally, in absence of data from other countries of the world, available data from Germany showed that the rate of infection was increasing among people aged 21–40 years—however, decreasing among all other age groups (Supplemental Figure S2). The number of COVID-19 cases and the number of deaths caused by COVID-19 both has been increasing up until the writing of this article (December 31, 2020) since the beginning of the pandemic; however, the number of deaths has not been increased at the same rate as the number of reported cases increased (Supplemental Figure S3).

## DISCUSSION

We performed three time-series models taking real-time data into consideration to detect global trends of daily or weekly reported COVID-19 CFR. We identified a declining trend since May 2020. Using the M–K trend test, we found an increasing trend for global daily rCFR values of COVID-19 until the 17th week (the pre-peak period, which ends on April 28, 2020) at 7.23%, and subsequently a significant declining trend up until the 53rd week (the post-peak period) to 2.2% (December 29–31, 2020). Using a more robust time series model (ARIMA, Prophet, and SES), we detected a strong declining trend of COVID-19 rCFR. Amongst three time-series models, the ARIMA model outperformed the benchmark SES and Prophet models, which is probably because the SES and Prophet methods were originally developed to handle business-related problems.^19,32^

The rCFR of COVID-19 was associated with different factors, of which the percentage of people aged 65 and above, and the prevalence of obesity were both strong predictors. This is a narrow variable set; and other factors, such as median age of diagnosed people in each country, innate population immunity, latitude of the country, or prevalence of vitamin D deficiency could also be possible drivers but were not included here.^5^ Our findings of declining rCFR trends is consistent with findings from hospital-based studies using data of the early and later phases of pandemic data.^45,46^ In New York, the mortality rate among hospitalized patients decreased by 18–20% in a 3 to 4 month period, accounting for 25.6% in March and 7.6% in June 2020.^45^ In England, the mortality rate at the Intensive Care Unit and High Intensive Unit decreased substantially among the patients admitted in May, compared with those admitted in March (9% and 11.2%, respectively).^46^

The rCFR is decreasing gradually over time, and the exact reason for this decrease is beyond the remit of this study. However, the decreased rCFR could be attributed to several reasons, such as the following: increased numbers of asymptomatic or mild cases being detected by widespread rollout of testing, introduction of dexamethasone and other improvements in medical management of severely ill patients, experience gained by health professionals, increased public awareness, shielding from infection, possible effects of repurposed drugs such as ivermectin that are increasingly used empirically, or increased rates of infection in younger people who have favorable outcomes, and shielding of people with co-morbidities.^10,47,48^

Globally, the COVID-19 cases are increasing, with more than 200,000 daily cases from July 21, 2020 to up until the writing of this article (December 31, 2020). However, rCFR is decreasing after April 28, 2020. The decreasing of COVID-19 rCFR could be partly anomalous with the increasing number of COVID-19 tests,^3^ which allows detection of more mild and asymptomatic cases that prior to this were excluded. For example, in Germany, the mean number of daily tests was 22,829 in the months of April 2020, and the figure was 117,423 in August 2020.^4^

Our analysis confirms that the declining trend in rCFR is not merely associated with increased COVID-19 testing. In our estimation, before the peak mortality period, an increase of 1,000 COVID-19 tests decreased the rCFR by 2%. However, during the post-peak period, an increase of a similar number of samples tested decreased the rCFR by 1%—but this is not statistically significant (*P* = 0.14). During this period, other variables, especially the percentage of people aged 65 and above, had a significant influence on the rCFR. An increase of 1% of a population above 65 years increased the rCFR by 1%, and an increase of 1% of an obese population increased the rCFR by 1%. Our results on obesity and its correlation with increasing rCFR are confirmed by other research on individual patients. Research showed that being overweight and obesity were risk factors for serious illness and these patients were more likely to experience complications such as respiratory failure and acute respiratory distress syndrome.^8,49^ The inverse relationship between GHSI and rCFR (or mortality rate) is discussed in earlier studies,^50,51^ which is consistent with our findings. Countries with a higher GHSI score have reported higher rCFR in recent data, and the exact reasons for this are speculated to include general poor health of the populations as measured by comorbidities, age prevalence, and other factors such as complacency and late response times to the lockdown process. The seriousness of the illness among those infected has overwhelmed healthcare systems and frontline healthcare providers in many of these higher GHSI countries and has drained resources, exposing how ill-equipped the world was to handle the pandemic.^51,52^ However, outbreak settings often generate incomplete data, where both recovered and fatal cases go unreported.

The declining global rCFR could be associated with several other factors, including improvements in health care management. For example, dexamethasone, a corticosteroid, was shown to save lives for patients with COVID-19’s severe acute respiratory syndrome. In the case of patients on ventilators, treatment with dexamethasone reduced the death rate by about one-third; and for patients who needed oxygen, the death rate was reduced by about one-fifth.^10,53^ Furthermore, two anti-inflammatory drugs (tocilizumab and sarilumab) showed some beneficial effects while used in intensive care unit patients.^9^ Compared with the placebo group, the drugs could reduce deaths by one-quarter.^9^ However, this result has not been replicated in other studies.^54^ Furthermore, most of the countries improved their ability to support uninterrupted high-flow nasal oxygen support for patients developing acute respiratory distress syndrome, a technique that could help to reduce mortality.^55,56^ Similarly, systemic anticoagulants were associated with beneficial effects on the survival of mechanically ventilated patients suffering from severe COVID-19 pneumonia.^11^

Our findings of the proportion of elderly people being at risk for higher rCFR is consistent with previous findings.^3,5,51^ Elsewhere, the risk of death was recorded as 13- to 73-fold lower in nonelderly people (< 65 years) than in older individuals.^57^ Another study showed that people above 65 years of age represent 80% of hospitalizations with COVID-19 and have a 23-fold greater risk of death than those under 65.^58^ Older people surviving with comorbidities common in technically advanced societies possess relatively compromised immune systems and are more vulnerable to infectious disease.^59^ The rate of infection in younger people is increasing globally (we have presented the data from Germany^44^ only; however, the pattern is consistent in other countries as well^60^). Younger people aged below 40 years in the United States, Israel, and Portugal appeared to be the main group of new cases.^60,61^

Although our analysis indicates that global rCFR because of COVID-19 is declining, it does not mean that the rCFR is decreasing in every country, and it should not be confused with Infection Fatality Rate or IFR (in other words, a lower risk of dying when being infected). In many countries, rCFR remains high and/or is rising. For example, in Yemen,^62^ a country with a fragile health system, is experiencing a rCFR above 28.9% as of December 31, 2020, when the global rCFR is estimated at 2.20%.^4^ Our findings also do not indicate that the virus is becoming less severe. A study on genetic characteristics of SARS-CoV-2 indicates that the virus had a mutation with the G614 spike, which has replaced D614 and has become the dominant variant of the virus around the world.^63^ The mutation is likely associated with increased infectivity; however, the pathogenicity of the variant remains unknown.^63^ More research is needed to measure the host-level pathogenicity of the virus.

## LIMITATION

We collected publicly available COVID-19 data on reported tests, cases, and deaths from WHO and other sources. These publicly available data probably contain under-reported values both in the numerator (COVID-19 deaths) and denominator (COVID-19 cases). There are variations in capacities and readiness of countries in testing and reporting COVID-19 cases and mortality records, which might have affected overall data quality. We estimated the cumulative rCFR, which tends to underestimate the actual risk of death because the deaths that will occur in the future are not included in the dataset. Both are universal limitations of rCFR estimated being used in most of the studies using global COVID-19 data. One of the key hypotheses is that a higher proportion of younger populations are being infected with COVID-19 at the later phase of the pandemic. However, we could not test whether the median age of the population is changing over time and whether these changes are contributing to lowering the rCFR. This study shows a declining rate of rCFR, but our data cannot determine if IFR might be declining as well.

## CONCLUSION

The global cumulative reported case fatality rate (rCFR) of COVID-19 increased up until the 17th epidemiological week (April 22–28, 2020) and then started to decline steadily. We found a negative association between the increasing number of tests and a decreasing rate of rCFR for COVID-19. Although increased tests help identification of more asymptomatic and mild cases, our analysis showed that the number of tests has a low impact on rCFR, especially during the post-peak period (weeks 18 to 53). The rCFR of COVID-19 was strongly associated with the percentage of people aged 65 and above in addition to the prevalence of obesity in the country. Exact reasons for lowering rCFR need to be studied more in detail but could possibly be explained by an increased rate of infection in younger people, by an improvement of health care management, by drugs that could reduce the mortality outcome and hospital stay of COVID-19 patients, or shielding of peoples with co-morbidities. This study supports a growing consensus on risk factors associated with CFR from different national datasets and experiences of the pandemic. Further studies are needed to understand the pattern of COVID-19 rCFR and host-level pathogenicity of the virus.

## Supplemental figure

Supplemental materials
