# Supplementary material for: The Global Case-Fatality Rate of COVID-19 Has Been Declining Since May 2020
Source: Am J Trop Med Hyg. 2021 Apr 21;104(6):2176–84. doi: 10.4269/ajtmh.20-1496 (PMC8176487; doi:10.4269/ajtmh.20-1496)

The following are supplemental files and will be available online only

**Supplemental Fig S1:** The top 10-countries with COVID-19 CFR before (January 1st – April 28^th^ 2020) and after peak (April 29 -- December 31st 2020) mortality period. Countries with more than 1 million population are included.


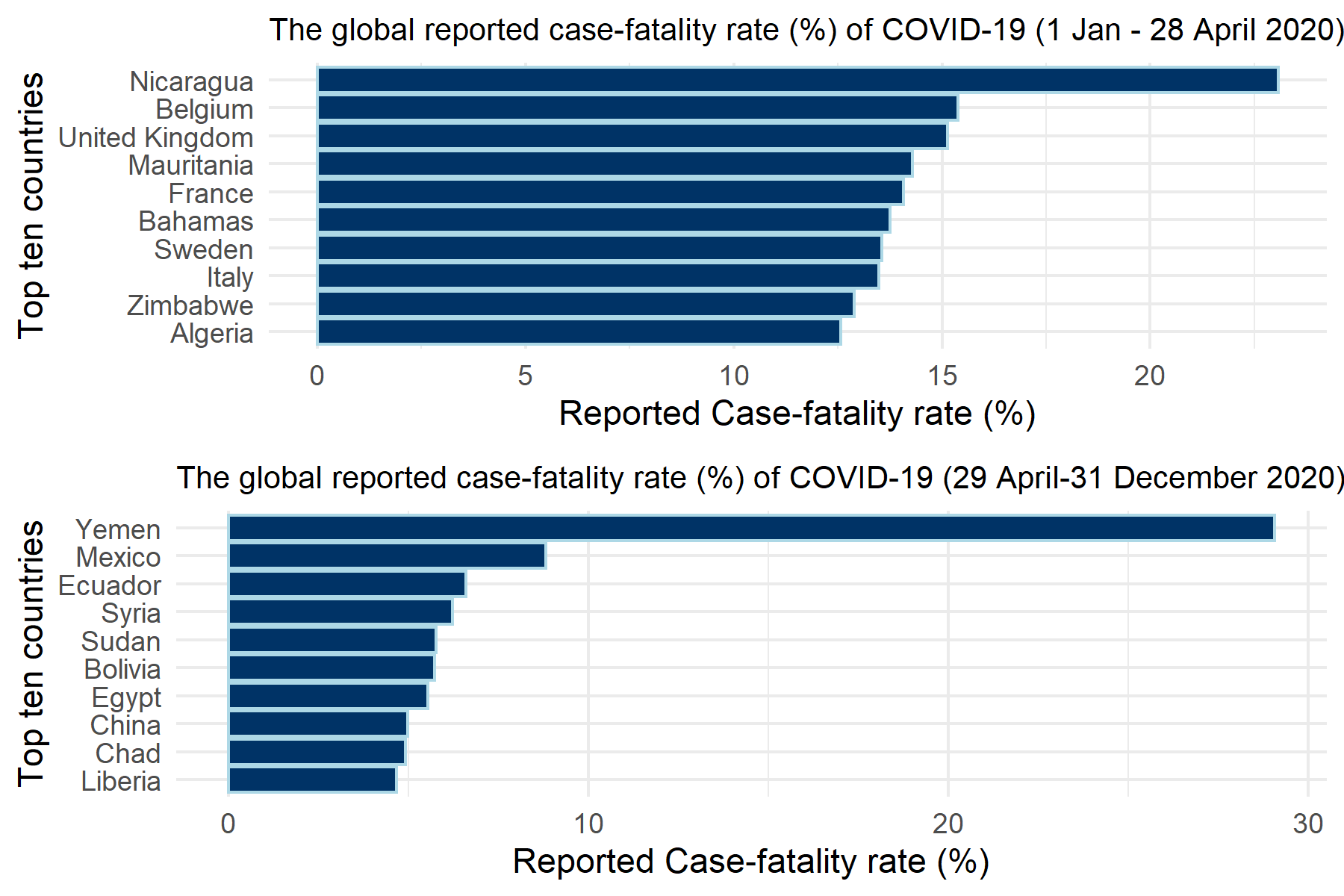


**Supplemental Fig S2:** The rate of COVID-19 infection over time (1st March – December 31st 2020) in Germany (details data on age group from other countries are not publicly available). Although rate of infection is decreasing in most age group, the rate increased among people aged 21--40 years up until November, 2020. However, from December an older people aged 80 years and above started to get infection at a higher rate and rate in younger people (< 40 years) started to decline.


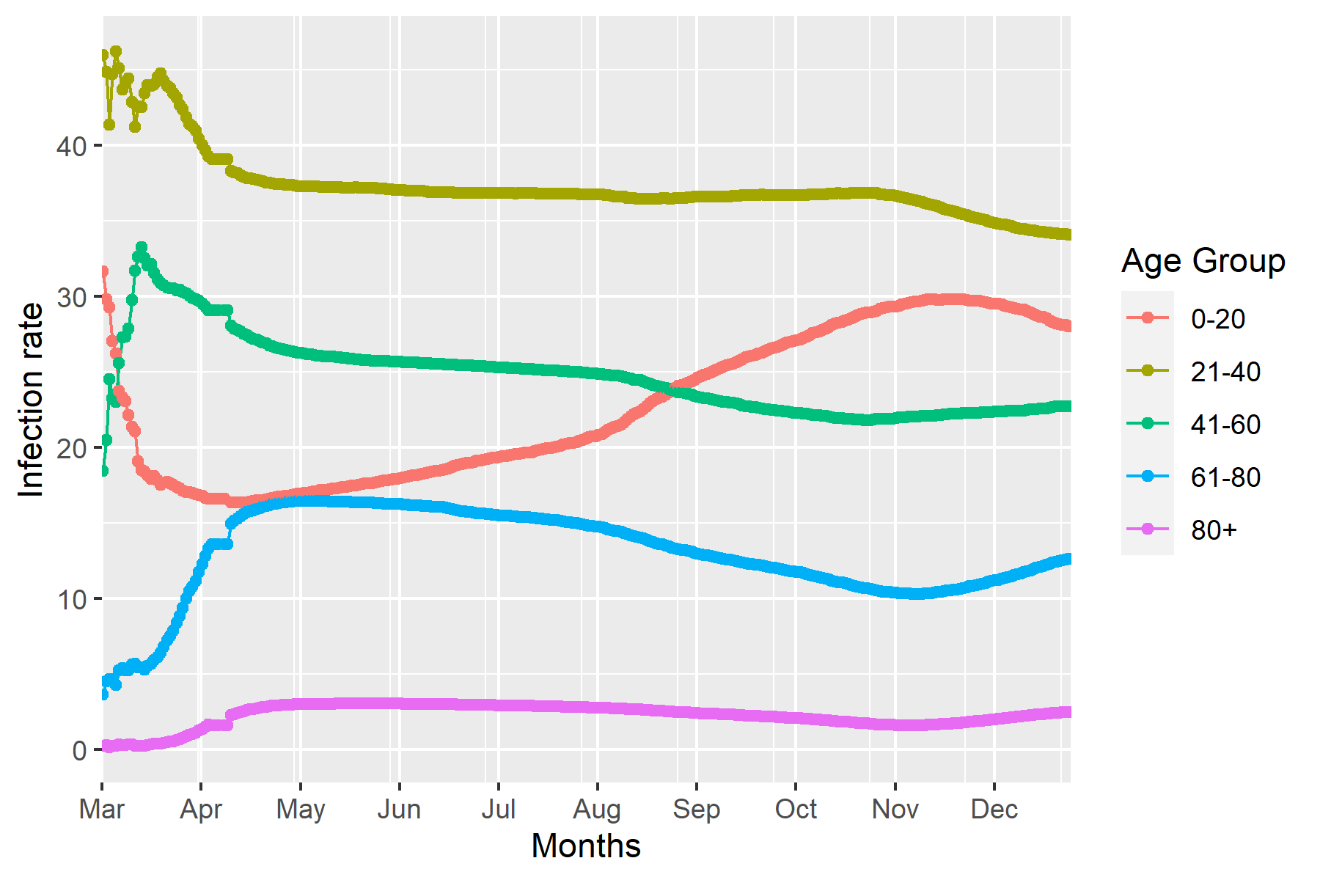


**Supplemental Fig S3:** The monthly number of new COVID-19 cases and new recorded deaths due to COVID-19 reported globally. The number of cases and deaths has been increasing consistently since beginning of the pandemic up until writing of this manuscript (31 December 2020). However, the number of deaths has not increased as the same rate of new cases increased.


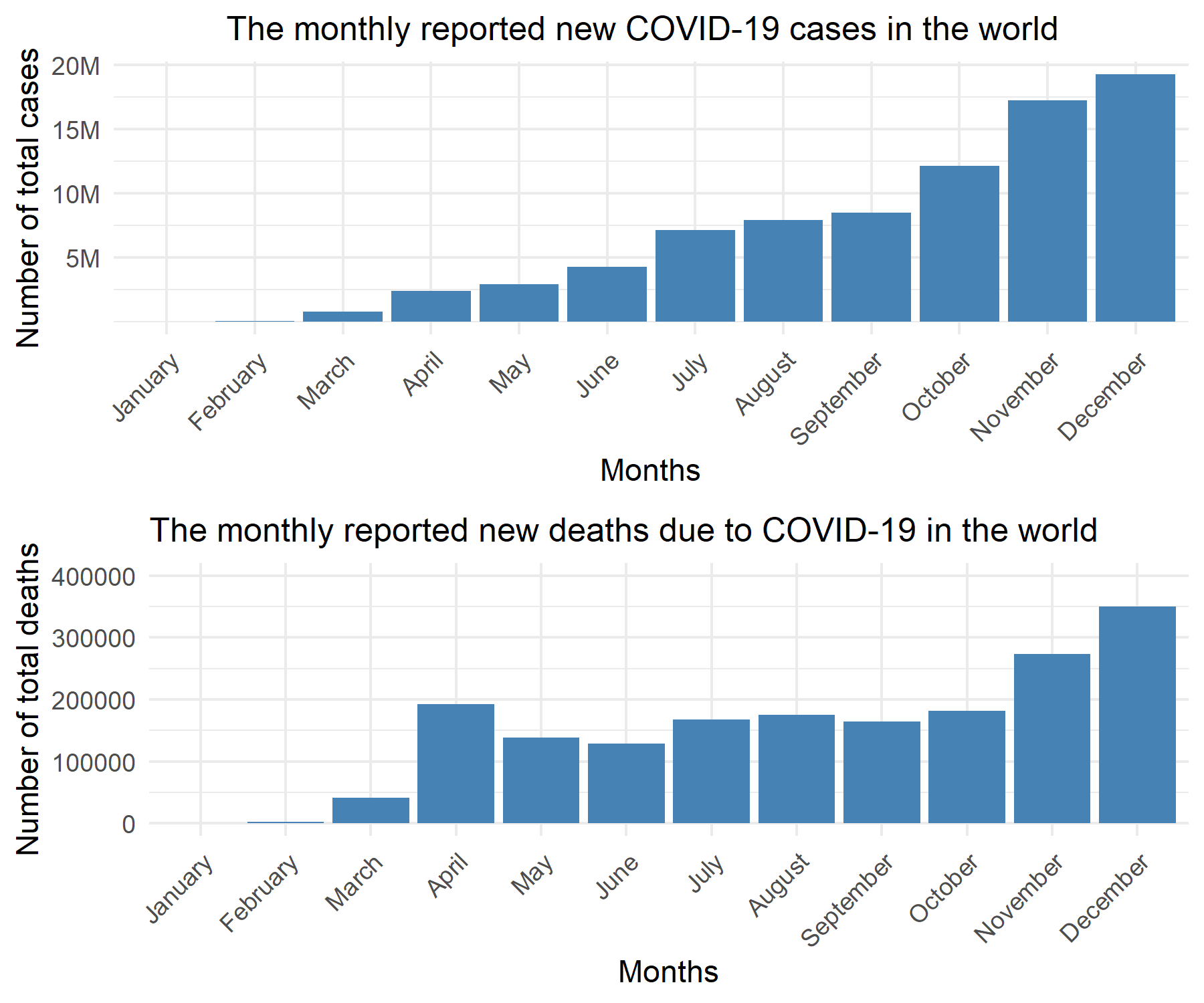

Supplement: Supplementary file 1 [file tpmd201496.SD1.docx]
